# Cass County, (Mich.) Medical Society

**Published:** 1851-09

**Authors:** D. E. Brown, A. Garwood

**Affiliations:** President; Cassapolis; Secretary; Cassapolis


### *Cass County (Mich.) Medical Society.*

The Medical Society met pursuant to adjournment. Dr. David E. Brown was called to the Chair, and Dr. A. Garwood was appointed Secretary.

The Society adopted a Consttution, By-Laws, code of Medical Ethics and a Fee Bill, which were reported by Committees that were appointed at a previous meeting.

The following members were present at the organization: Drs. D. E. Brown, J. Allen, E. H. Keables, L. Tompkins, E. Reading, B. Wells, H. Lockwood, A. Garwood, E. Penwell, J. G. Bugbee, E. J. Bonine, T. Brayton, and L. K. Raymond.

After the above named Physicians signed the Constitution, on motion of Dr. H. Lockwood, the Society proceeded to elect its officers, who were chosen as follows, viz:

President, Dr. D. E. Brown; Vice President, Dr. H. Lockwood; Secretary, Dr. A. Garwood; Treasurer, Dr. E. Penwell; Standing Committee, Drs. J. G. Bugbee, J. Allen, and B. Wells.

On motion of Dr. Wells, it was

*Resolved*, That the proceedings of this meeting be published in the National Democrat and North Western Advocate.

On motion of Dr. Bugbee, it was

*Resolved*, That the proceedings be published in the Boston Medical and Surgical Journal, and in the North-Western Medical and Surgical Journal.

On motion of Dr. H. Lockwood, the Society adjourned to meet on the first Monday in November next, at Edwardsburg, at 10 o'clock A. M.

D. E BROWN, *President*.

A. GARWOOD, Secretary.

*Cassapolis*, *Aug*. 14, 1851.

